# Supplementary material for: Sorting at embryonic boundaries requires high heterotypic interfacial tension
Source: Nat Commun. 2017 Jul 31;8:157. doi: 10.1038/s41467-017-00146-x (PMC5537356; doi:10.1038/s41467-017-00146-x)
Supplement: Supplementary file 2 — Supplementary Software 1 [file 41467_2017_146_MOESM2_ESM.zip › PottsModel/SrcPottsModel/doc/model/class-use/IPositionManager.Position.html]

Uses of Class model.IPositionManager.Position


JavaScript is disabled on your browser.


Skip navigation links


- Overview
- Package
- Class
- Use
- Tree
- Deprecated
- Index
- Help

- Prev
- Next

- Frames
- No Frames

- All Classes

## Uses of Class model.IPositionManager.Position

- Packages that use IPositionManager.Position

  | Package | Description |
  | --- | --- |
  |  |  |
  | --- | --- |
  | gui |  |
  | model |  |
- - ### Uses of IPositionManager.Position in gui

    Methods in gui with parameters of type IPositionManager.Position

    | Modifier and Type | Method and Description |
    | --- | --- |
    |  |  |
    | --- | --- |
    | `Hexagon.CubeCoordinates` | Hexagon.CubeCoordinates.`getDiagonalNeighborCoordinates(IPositionManager.Position p)` |
  - ### Uses of IPositionManager.Position in model

    Fields in model declared as IPositionManager.Position

    | Modifier and Type | Field and Description |
    | --- | --- |
    |  |  |
    | --- | --- |
    | `static IPositionManager.Position[]` | IPositionManager.`HexagonPositions` |
    | `static IPositionManager.Position[]` | IPositionManager.`SquarePositions` |

    Methods in model that return IPositionManager.Position

    | Modifier and Type | Method and Description |
    | --- | --- |
    |  |  |
    | --- | --- |
    | `IPositionManager.Position[]` | SquarePositionManager.`getAllNeighborPositions()` Returns left, bottom, right and top positions (in this order). |
    | `IPositionManager.Position[]` | IPositionManager.`getAllNeighborPositions()` Defines neighbor positions for given position model. |
    | `IPositionManager.Position[]` | HexagonPositionManager.`getAllNeighborPositions()` Returns top, topRight, bottomRight, bottom, bottomLeft, topLeft positions (in this order). |
    | `IPositionManager.Position[]` | SquarePositionManager.`getConnectedNeighborPositions()` |
    | `IPositionManager.Position[]` | IPositionManager.`getConnectedNeighborPositions()` |
    | `IPositionManager.Position[]` | HexagonPositionManager.`getConnectedNeighborPositions()` |
    | `IPositionManager.Position[]` | SquarePositionManager.`getPartialNeighborPositions()` |
    | `IPositionManager.Position[]` | IPositionManager.`getPartialNeighborPositions()` Returns partial neighbors so that iterating over all pixels and getting their respective partial neighbors will only result in passing over them a single time. |
    | `IPositionManager.Position[]` | HexagonPositionManager.`getPartialNeighborPositions()` |
    | `static IPositionManager.Position` | IPositionManager.Position.`valueOf(java.lang.String name)` Returns the enum constant of this type with the specified name. |
    | `static IPositionManager.Position[]` | IPositionManager.Position.`values()` Returns an array containing the constants of this enum type, in the order they are declared. |

    Methods in model with parameters of type IPositionManager.Position

    | Modifier and Type | Method and Description |
    | --- | --- |
    |  |  |
    | --- | --- |
    | `Pixel` | SquarePositionManager.`getNeighborFromPosition(Pixel pPixel, IPositionManager.Position pPosition)` |
    | `Pixel` | IPositionManager.`getNeighborFromPosition(Pixel pPixel, IPositionManager.Position pPosition)` |
    | `Pixel` | HexagonPositionManager.`getNeighborFromPosition(Pixel pPixel, IPositionManager.Position pPosition)` |

Skip navigation links


- Overview
- Package
- Class
- Use
- Tree
- Deprecated
- Index
- Help

- Prev
- Next

- Frames
- No Frames

- All Classes
